# Behavioral Teleporting of Individual Ethograms onto Inanimate Robots: Experiments on Social Interactions in Live Zebrafish

**DOI:** 10.1016/j.isci.2020.101418

**Published:** 2020-07-29

**Authors:** Mert Karakaya, Simone Macrì, Maurizio Porfiri

**Affiliations:** 1 Department of Mechanical and Aerospace Engineering, New York University, Tandon School of Engineering, 6 MetroTech Center, Brooklyn, NY 11201, USA; 2 Centre for Behavioural Sciences and Mental Health, Istituto Superiore di Sanità, Viale Regina Elena 299, 00161 Rome, Italy; 3 Department of Biomedical Engineering, New York University, Tandon School of Engineering, 6 MetroTech Center, Brooklyn NY 11201, USA

**Keywords:** Biological Sciences, Ethology, Robotics

## Abstract

Social behavior is widespread in the animal kingdom, and it remarkably influences human personal and professional lives. However, a thorough understanding of the mechanisms underlying social behavior is elusive. Integrating the seemingly different fields of robotics and preclinical research could bring new insight on social behavior. Toward this aim, we established “behavioral teleporting” as an experimental solution to independently manipulate multiple factors underpinning social interactions. Behavioral teleporting consists of real-time transfer of the complete ethogram of a live zebrafish onto a remotely-located robotic replica. Through parallel and simultaneous behavioral teleporting, we studied the interaction between two live fish swimming in remotely-located tanks: each live fish interacted with an inanimate robot that mirrored the behavior of the other fish, and the morphology of each robot was independently tailored. Our results indicate that behavioral teleporting can preserve natural interaction between two live animals, while allowing fine control over morphological features that modulate social behavior.

## Highlights

• The ethogram of a live fish is transferred onto a remotely-located robotic replica
• Social interactions of two zebrafish of different size are experimentally studied
• Behavioral teleporting allows fine control over morphological features
• The qualitative and quantitative nature of social interactions can be preserved

## Graphical Abstract

**Figure:**
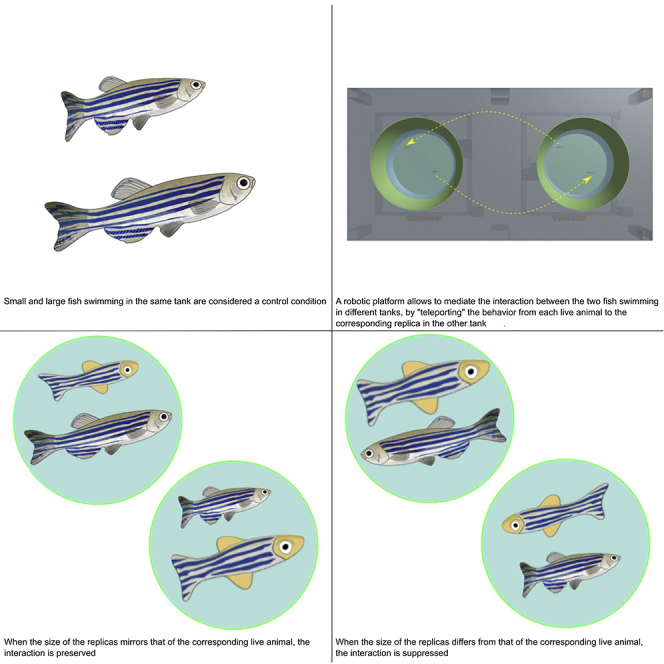

## Introduction

Social behavior determines the survival and reproductive success of many organisms (Choe, 2019). In humans, social behavior unfolds in actions, habits, and practices that ultimately define our individual life and our society (Bandura and Walters, 1977). Major deviations from societal norms often constitute symptoms of psychiatric disorders (autism [Lord et al., 2000] and schizophrenia [Buckner et al., 2008]). Inter-individual interactions are the outcome of complex processes, mediated by individual traits (genetic or environmental) and behavioral feedback that are often difficult to tease out from each other. For example, upon entering a meeting or approaching an unknown individual, we form strong biases within milliseconds (Naumann et al., 2009; Willis and Todorov, 2006). Factors like baldness, height, voice pitch, and outfit are likely to skew our consideration of other people (Nelissen and Meijers, 2011). As a result, behavioral feedback by our peers could have a very different effect, as a function of their appearance. How can we discriminate the effects of appearance and behavioral feedback? And, which visual features really matter?

Addressing these questions demands the isolation of the potential intervening variables through hypothesis-driven experiments. Laboratory animal studies represent one of the primary tools in this realm. Although rodents have traditionally constituted the taxa of choice, zebrafish are now emerging as a valid alternative (Macrì and Richter, 2015). This freshwater species is amenable to disentangling gene × environment interactions, whereby their genome is fully sequenced and modifiable through gene-editing procedures (Fontana et al., 2018; Kalueff et al., 2014). Likewise, pharmacological treatments targeting a given biological pathway are widely documented and procedurally standardized (Bao et al., 2019; de Abreu et al., 2019). Within the field of social interactions, zebrafish have been employed to investigate both normal behaviors and abnormal derailments (Meshalkina et al., 2018; Morris, 2009). Tang and collaborators (Tang et al., 2020) recently conducted a comprehensive study investigating the role of genes, putatively involved in human psychiatric disorders, in social behavior. Specifically, the authors tested 90 mutant lines against wild-type zebrafish and observed that the aforementioned mutations may alter the collective behavior of adult individuals. This study combined genetic engineering with a detailed automated phenotyping and advanced statistics to demonstrate that genetic manipulations map onto three identifiable patterns of collective behavior: group cohesion, alignment, and density. Yet, genetic engineering alone cannot provide the fine degree of control that is needed to determine the mechanism underlying social behavior.

For example, while addressing whether a specific gene or treatment (be it pharmacological or environmental) is involved in sociability, it is necessary to test how the experimental subject responds to a “standard” opponent. The “standard” nature of these opponents is far less than standardized—for rigorous scientific reasons. Standard opponents, in social interaction tests, may take the form of wild-type vehicle-treated control subjects (Kim et al., 2017; Liu et al., 2018) or subjects with a heterozygous background (Liu et al., 2018). Regardless of the selection of the most appropriate control group, even genetic manipulations per se may beget spurious phenotypes (Kim et al., 2017). Albeit extremely selective in nature, genetic manipulations have been shown to co-occur with off-target effects (genetic compensation), for which precise mechanisms have remained elusive (El-Brolosy and Stainier, 2017). Limited selectivity of the target phenotype extends beyond genetics, whereby the administration of psychoactive substances may influence many independent factors like locomotory patterns (Ladu et al., 2014), visual cues (Gerlai et al., 2000), and hormonal secretion (Sterling et al., 2015). Under these conditions, attributing the modulatory effects of a given drug on sociality to a specific underlying mechanism becomes questionable.

To isolate appearance from behavioral feedback in social interactions, several authors have proposed the integration of engineered, artificial stimuli in the form of visual playback or computer-animated images (Gerlai, 2010, 2014; Woo and Rieucau, 2011). Although these stimuli can attain a great degree of visual similarity, their locomotory patterns could inherently suffer from an imprecise representation of the real-life stimulus they were meant to resemble. The locomotion of visual images projected on computer screens is, by definition, constrained to the two-dimensional realm, which may fail to offer a realistic stimulus to live zebrafish. Recently, virtual reality has been explored as a powerful approach to create a rich, three-dimensional representation of the social environment (Naik et al., 2020; Stowers et al., 2017). Virtual reality overcomes several limitations of video playback and computer-animated images, by considerably enriching the repertoire of interactive behaviors that could be engineered and by improving the immersiveness of the experience by focal subjects. Besides coming at a considerable and potentially unaffordable cost that may challenge its widespread use in preclinical research, this approach still relies on projected images that may not fully capture the complexity of the three-dimensional stimulus of a live conspecific.

Biologically-inspired robots offer a promising alternative to virtual reality, by affording the delivery of physical, easily controllable, three-dimensional stimuli (Abdai et al., 2018; Frohnwieser et al., 2016; Porfiri, 2018; Romano et al., 2019). Yet, in comparison with virtual reality, robotics-based experimental setups do not allow an analogous level of controllability over morphological features and locomotory patterns. Additionally, it could be difficult to create an equivalently immersive experience through robotics-based setups, which are constrained by the practical limitations associated with any mechanical hardware. Despite these limitations, biologically-inspired robotic stimuli constitute faithful, three-dimensional representations of conspecifics in laboratory experiments on social behavior.

Although the initial efforts in this area were robotic stimuli that could only move along *a priori* determined trajectories, recent studies have demonstrated the possibility of interactive experiments with behavioral feedback (Bonnet et al., 2016; Cazenille et al., 2018; De Lellis et al., 2020; Kim et al., 2018; Kopman et al., 2013; Landgraf et al., 2013, 2016; Papaspyros et al., 2019). In all these interactive robotic platforms, an external manipulator is utilized to maneuver biologically-inspired replicas that interact with live animals on the basis of real-time feedback from an automated tracking system. The main difference among these robotic platforms is in the approach that is pursued to control the movements of the replica in response to the behavior of live animals. Some studies have proposed the integration of classical behavioral rules inspired by mathematical models of schooling and shoaling (Bonnet et al., 2016; Kim et al., 2018; Kopman et al., 2013; Landgraf et al., 2013, 2016), and others have employed recent data-driven stochastic models of fish behavior (De Lellis et al., 2020; Papaspyros et al., 2019) or alternative probability-based feedback mechanisms (Cazenille et al., 2018).

Notwithstanding their contribution to fundamental science and the corresponding level of technological sophistication, existing robotic platforms often lead to unnatural behavioral responses of live animals. We propose that this limited degree of biomimicry is associated with the common premise of all these approaches to use a mathematical representation of social behavior for controlling the movements of the replica. To obviate the need of a mathematical representation of social behavior, we put forward the concept of “behavioral teleporting.” Within behavioral teleporting, we transfer, in real time, the behavior of a live zebrafish onto the movements of a biologically-inspired replica located in another tank. The “copy/pasted” behavior of the live fish replaces the mathematical representation on which existing approaches are based.

Our concept of behavioral teleporting is related to two recent, independent breakthroughs by Bonnet and collaborators (Bonnet et al., 2019) and Larsch and Baier (Larsch and Baier, 2018). Bonnet and collaborators demonstrated the feasibility of remote interaction between zebrafish and honeybees in binary decision-making tasks through biologically-inspired robots (a zebrafish replica and two bee-robots). The authors showed that, by controlling the zebrafish replica through spatial density of honeybees and the bee-robot through the swimming direction of zebrafish, it was possible to establish a link between these two species. Larsch and Baier demonstrated the possibility of establishing remote social interactions between zebrafish within a virtual reality setup, wherein projected dots instantaneously replicated the motion of independent subjects located in different tanks. Our approach combines these two strategies by affording the transfer of the complete ethogram of a zebrafish, similar to Larsch and Baier (2018), onto a three-dimensional robotic replica, similar to Bonnet et al. (2019).

**Figure 1:**
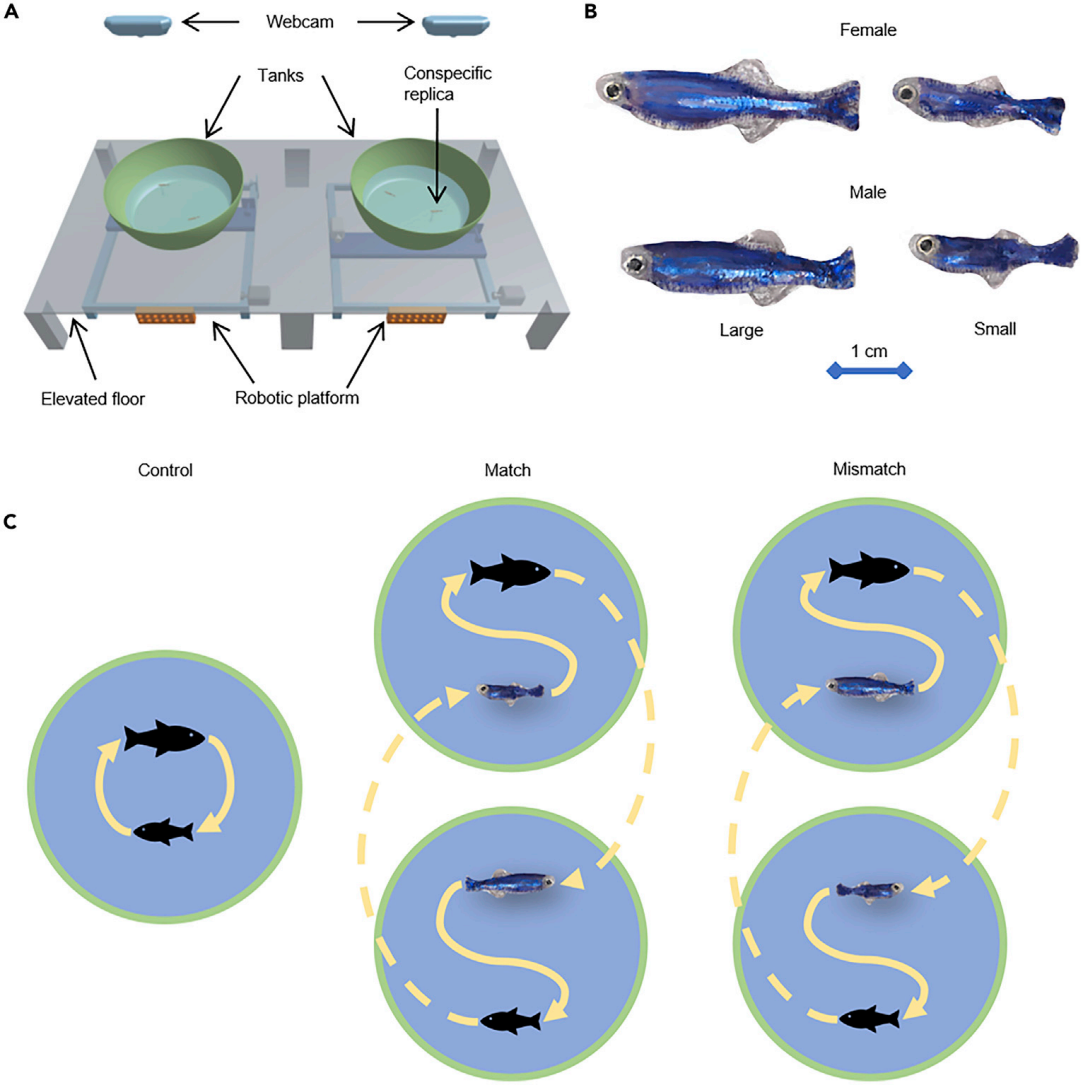
Experimental Setup and Conditions For a Figure360 author presentation of Figure, see https://doi.org/10.1016/j.isci.2020.101418. (A) Experimental apparatus with two robotic platforms, two tanks, the base structure, and two overhead cameras. (B) Female and male conspecific replicas of large and small sizes. (C) Experimental conditions considered in our study: Control, Match, and Mismatch. The black cartoon fish represent live fish. Interactions within the same tank are highlighted through solid lines, whereas dashed lines indicate behavioral teleporting of a live fish onto a replica in a separate tank.

To validate the efficacy of our approach in the study of social interactions, we implemented behavioral teleporting in a setup consisting of two separate tanks, each containing one fish and one robotic replica (Figure 1A). An automated tracking system scored, in real time, the locomotory patterns of each of the live subjects, which were used to control the robotic replica swimming in the other tank. The complete ethogram of each fish was transferred across tanks within a fraction of a second, thereby establishing a complex robotics-mediated interaction between two remotely-located live animals.

The relevance of our approach to unveil the mechanism of social behavior was tested in an ethologically meaningful experiment, in which we systematically varied behavioral feedback and appearance (Figure 1B). We investigated whether behavioral teleporting would preserve the interaction between a large and a small fish, provided that the morphology of the robotic replicas resembled their live counterparts. To confirm this association, we manipulated the morphology of the replicas, such that a live subject would swim with a replica resembling a small but behaving like a large fish or looking large but behaving small (Figure 1C).

## Results

### The Ethogram of a Live Zebrafish Can Be Transferred through Behavioral Teleporting

**Figure 2:**
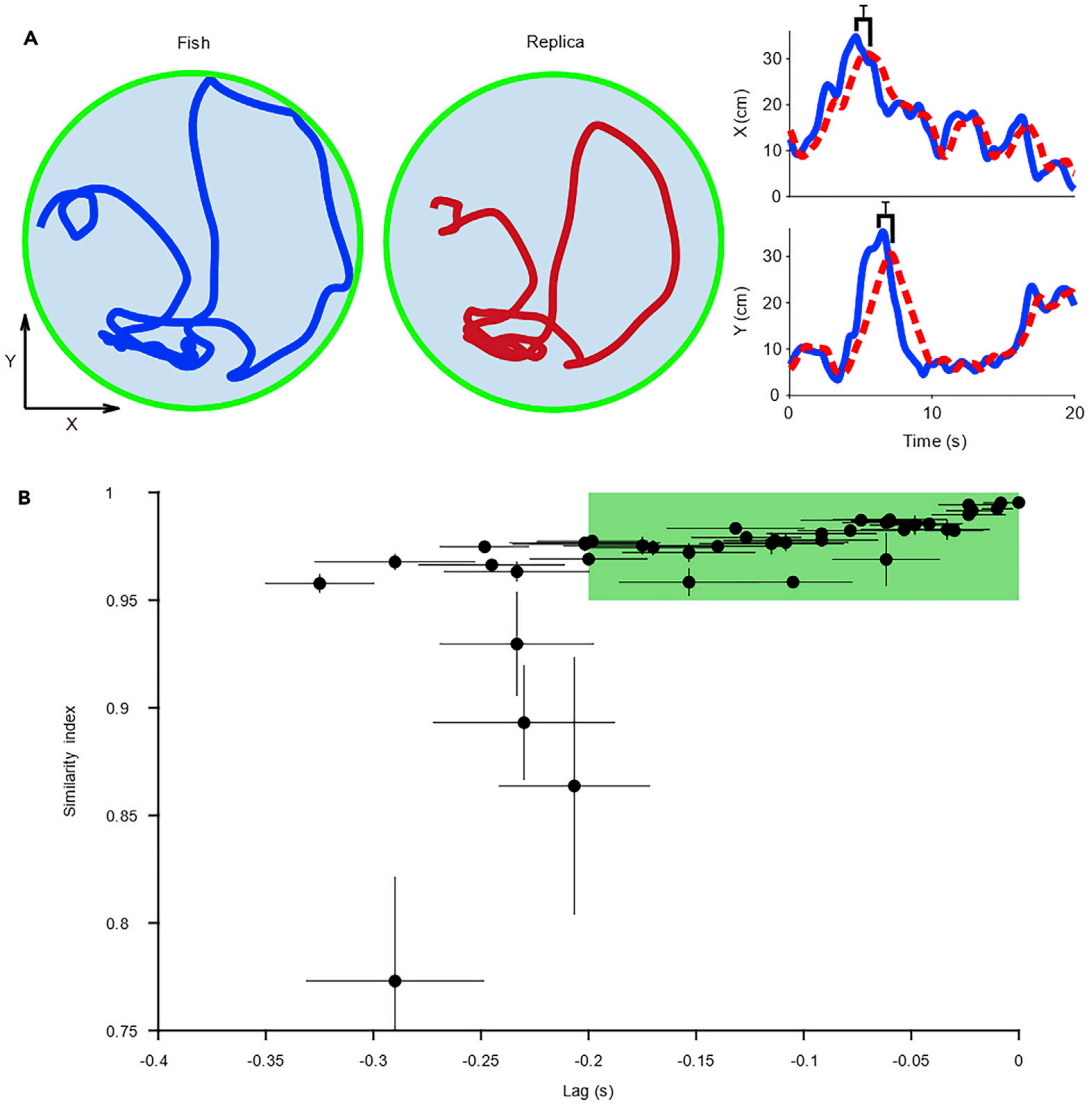
Performance of the Robotic Platform (A) Left panel: trajectory of a small fish in Match condition over a 20-s time interval. Center panel: trajectory imposed on the small replica teleporting the behavior of the small fish in Match condition over the same time interval. Right panel: X and Y coordinates of the fish (blue line) and replica (dashed red line); brackets show the time-lag required to attain the maximum normalized cross-correlation (similarity index) between the replica and the fish. (B) Similarity index as a function of the time-lag. For each experimental session, there are two circles that represent the averages of 30 similarity indices and corresponding time-lags. Whiskers represent standard deviations, and the green region identifies the range of successful experiments (22 trials/1,224 time intervals) in which the similarity index was above 0.95 within a time-lag of less than 0.2 s.

One of the primary aims of our work was to demonstrate the capability to transfer the motion of a live fish onto a robotic replica. To this end, we scored in real time the behavior of a live fish and, through an in-house developed robotic platform, we accordingly maneuvered a replica, located in a separate tank (Transparent Methods). Behavioral teleporting was always performed on two animals at the same time, thereby maneuvering two replicas at the same time. The extent to which a replica was successful in mirroring the motion of a live animal was assessed by cross-correlating their trajectories (Figure 2A). Experimental results indicate that the replica teleported the motion of the fish in almost all trials (85% of the total experimental time), with a 95% accuracy at a maximum time-lag smaller than 0.2 s (Figure 2B). The high accuracy in the replication of fish trajectory was confirmed by equivalent analysis on speed, turn rate, and acceleration (Text S1 and Figure S1).

In order to assess the feasibility of the developed system in hypothesis-driven experiments, we tested whether behavioral teleporting would preserve the simplest form of dyadic interaction between a large and small fish of the same sex (Figure 1C). Specifically, we compared the interaction between a small and a large fish swimming in the same tank against their remote interaction enabled by behavioral teleporting. In the Control condition, we scored the behavior of two live fish (one large and one small; average size difference of approximately 33%) of the same sex, swimming in the same tank for 10 min (Video S1). We pursued a within-subject experimental design, wherein the identity of every pair (12 pairs of fish of the same sex: six male pairs and six female pairs) was maintained across experimental conditions. Behavioral teleporting was implemented in the Match condition, where the same pairs tested in the Control condition were physically separated in two independent tanks. Within each tank, a fish of the pair swam with a conspecific-like replica matching the morphology and locomotory pattern of the fish located in the other tank (Video S2). As a result, the large fish swam with a small replica that mirrored the locomotory patterns exhibited by the small fish positioned in the other tank, and vice versa.

### Natural Patterns of Social Interactions Are Preserved in Match Condition

The interaction between the animals was scored through the information-theoretic notion of transfer entropy, which can be used to identify causal relationships between two systems in a Wiener-Granger sense (Bossomaier et al., 2016). We pursued a symbolic approach, in which we combined the time-series of the speed and turn rate to create a simplified, yet robust, representation of zebrafish ethogram. Four symbols were introduced based on the signs of the linear and angular accelerations; for example, one symbol described the case in which the speed of the fish decreased along with its turn rate. Computing transfer entropy between the symbolic time-series of the two fish in the same (Control condition) or different tanks (Match condition), we evaluated the extent to which knowledge about the behavior of one fish can improve the predictability of the future behavior of the other fish. Statistical significance was assessed through a non-parametric permutation test against a surrogate distribution of 20,000 randomly generated values. We expected to register transfer entropy values different from zero between the small and large fish in the Control condition, as they were allowed to swim in the same tank and mutually influence each other. Similarly, we predicted transfer entropy values different from zero in the Match condition, when the robotic replicas mediated the interaction between the subjects swimming in different tanks. Results in Table 1 confirm these predictions, pointing at a strong interaction between subjects swimming in the same or in different tanks (p < 0.001).

**Table 1:** Synoptic Presentation of the Transfer Entropy Results on the Interaction between Small and Large Zebrafish as a Function of the Experimental Conditions

	TE_Small→Large_	TE_Large→Small_	Net TE = TE_Small→Large_ - TE_Large→Small_
Control	6.504 × 10^−3^ (1.864 × 10^−3^) p < 0.001	5.896 × 10^−3^ (1.619 × 10^−3^) **p < 0.001**	6.073 × 10^−4^ (−2.076 × 10^−4^; 4.313 × 10^−4^) **p = 0.009**
Match	6.411 × 10^−3^ (1.935 × 10^−3^) **p < 0.001**	5.012 × 10^−3^ (1.521 × 10^−3^) **p < 0.001**	1.399 × 10^−3^ (−1.232 × 10^−4^; 6.344 × 10^−4^) **p < 0.001**
Mismatch	6.637 × 10^−3^ (2.169 × 10^−3^) **p < 0.001**	6.828 × 10^−3^ (2.178 × 10^−3^) **p < 0.001**	−1.901 × 10^−4^ (−3.837 × 10^−4^; 3.855 × 10^−4^) p = 0.129

Transfer entropy analysis: average values, 95% (2.5% and 97.5%) quantiles from surrogate distributions for one-tailed (two-tailed) tests, and p values. Bold p values indicate significant results at a 5% significance level in permutation tests.

**Figure 3:**
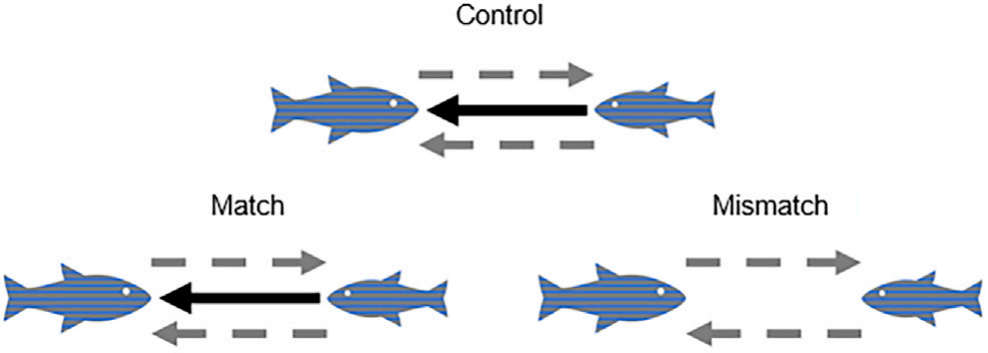
Graphical Representation of Significant Transfer Entropy Results across Conditions Dashed gray arrows represent significant transfer entropy from one fish to the other, and the solid black line identifies significant net transfer entropy. In Control and Match conditions, the small fish influences the large one more than the other way around, whereas in Mismatch condition the interaction is not significantly skewed. Transfer entropy is computed on the basis of the time-series of the speed and turn rate (Figure 4).

The robotic platform was not only successful in teleporting fish behavior across tanks and creating a remote interaction between subjects but also preserved the same relationship between small and large fish. We used net transfer entropy to quantify asymmetries in the pair, thereby detecting whether one of the two fish had a stronger influence on the other. In the Control condition, we observed that net transfer entropy was skewed toward the small fish (p = 0.009), thereby suggesting that the small had a stronger influence on the large than vice versa. The same skewness was preserved in the Match condition, wherein behavioral teleporting resulted in a stronger influence of the small fish on the large one swimming in a different tank (p < 0.001) (Figure 3). Delving into the qualitative nature of the asymmetric relationship between small and large fish, we investigated leader-follower relationships within the pair, following early work by Krause and collaborators who defined “leadership as the initiation of new directions of locomotion by one or more individuals which are then readily followed by other group members” (Krause et al., 2000). Within this realm, the fish that performed the first movement was considered the leader and the one that followed that movement was deemed a follower. We operationalized leader-follower interactions by computing cross-correlation on the time-series of either the speed or turn rate; an analogous approach to the quantification of leader-follower relationships has been used to study bird flocks (Nagy et al., 2010). For example, a leader would be defined as such if its accelerations or sudden turns were mirrored by the other fish (the follower). In practice, given one type of time-series (speed or turn rate), we computed the maximum value of the normalized cross-correlation and the corresponding time-lag. Although the value of the cross-correlation offers an indication of the strength of the interaction, the sign of the time-lag reveals which of the fish tends to lead the movement of the other (a positive value means that the small fish initiates movements that are followed by the large one, and a negative value refers to the opposite case). Similar to transfer entropy analysis, we assessed statistical significance through permutation tests against a surrogate distribution of 20,000 randomly generated values. We created a surrogate dataset by using the same approach as described for transfer entropy and analogously assessed statistical significance using a permutation test at 5% significance level.

**Figure 4:**
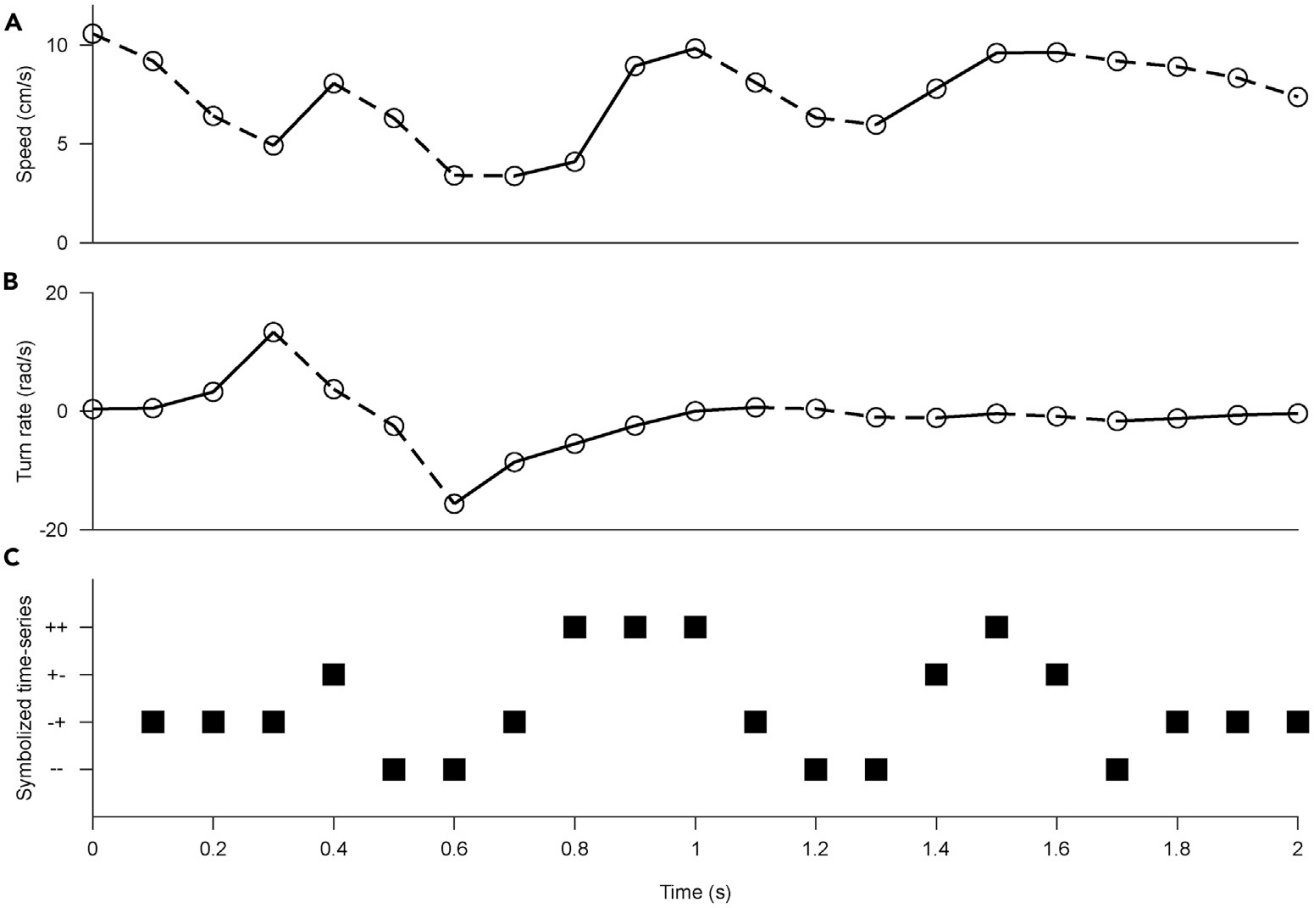
Joint Symbolization of Speed and Turn Rate (A) Time-series of the speed of a fish within a 2-s window. (B) Time-series of turn rate within the same window. Dashed and solid lines are associated with the decreasing and the increasing symbols, respectively. (C) Joint symbolic time-series with four symbols (“--” decrease in speed and turn rate; “-+” decrease in speed and increase in turn rate, “+-” increase in speed and decrease in turn rate, and “++” increase in both speed and turn rate).

Video S1. Exemplary Video of Two Live Fish Swimming in the Same Tank for the Control Condition, Overlaid with Tracking, Related to Figure 1

Video S2. Exemplary Video of Two Live Fish Swimming in Different Tanks with Robotic Replicas for the Match Condition, Overlaid with Tracking, Related to Figure 1

Video S3. Exemplary Video of Two Live Fish Swimming in Different Tanks with Robotic Replicas for the Mismatch Condition, Overlaid with Tracking, Related to Figure 1

With respect to speed in both Control and Match conditions, we registered values of normalized cross-correlation significantly larger than chance (normalized cross-correlation for Control and Match: 0.810 and 0.735, respectively; p < 0.001) with the small fish leading the large one significantly more often than chance (time-lag between small and large fish for Control and Match: 0.073 s and 0.057 s, respectively; p < 0.001) (Table 2). With respect to turn rate, Control subjects displayed value of the normalized cross-correlation significantly larger than chance (normalized cross-correlation: 0.241; p < 0.001), with the small fish leading the large one significantly more often than chance (time-lag between small and large fish: 0.077 s; p < 0.001) (Table 2). The same pattern was observed in Match subjects (time-lag between small and large fish: 0.037 s; p = 0.010), although the value of the normalized cross-correlation failed to reach statistical significance (normalized cross-correlation: 0.139; p = 0.422) (Table 2).

**Table 2:** Cross-Correlation Analysis on the Interaction between Small and Large Zebrafish for Different Experimental Conditions

	Normalized Cross-Correlation of Speed	Time-Lag for Speed	Normalized Cross-Correlation of Turn Rate	Time-Lag for Turn Rate
Control	0.810 (0.767) **p < 0.001**	0.073 (−0.008; 0.042) **p < 0.001**	0.241 (0.168) **p < 0.001**	0.077 (−0.025; 0.049) **p < 0.001**
Match	0.735 (0.723) **p < 0.001**	0.057 (−0.018; 0.027) **p < 0.001**	0.139 (0.141) p = 0.422	0.037 (−0.028; 0.031) **p = 0.010**
Mismatch	0.744 (0.740) **p < 0.001**	−0.025 (−0.029; 0.014) p = 0.052	0.142 (0.140) **p = 0.003**	0.022 (−0.025; 0.033) p = 0.096

Normalized cross-correlation and corresponding time-lags: average values 95% (2.5% and 97.5%) quantiles from surrogate distributions for one-tailed (two-tailed) tests, and p values. Bold p values indicate significant results at a 5% significance level in permutation tests.

### Natural Patterns of Social Interactions Are Altered in Mismatch Condition

To demonstrate that both morphology and locomotory patterns contribute to the relationship between the fish, we devised a condition in which the appearance of the replica was conflicting with its expected behavior. In this Mismatch condition, the behavior of a small fish was teleported onto a large replica swimming together with a large fish, whose behavior was, in turn, teleported onto a small replica swimming with the small fish (Figure 1C; Video S3). As mentioned above, the fish pairs identities were maintained throughout all conditions, so that the pairs tested in the Mismatch condition were the same as those tested in Control and Match conditions. Similar to Match condition, we registered a strong interaction between subjects swimming in the different tanks (p < 0.001) (Table 1). However, in accordance with our predictions, decoupling locomotory patterns from morphology abolished the asymmetry in the interaction, whereby net transfer entropy was undistinguishable from chance (p = 0.129) (Figure 3 and Table 1). It is important to emphasize that behavioral teleporting, together with the presence of replicas, did not influence general locomotion of live fish, whereby Control, Match, and Mismatch individuals exhibited indistinguishable values and temporal patterning of speed, turn rate, and acceleration, alongside with highly comparable spatial distribution in the experimental tank (Text S2 and Figures S2 and S3).

Delving into the qualitative nature of the interaction through cross-correlation analysis, we identified cross-correlation significantly larger than chance for both speed and turn rate (normalized cross-correlation: 0.744 and 0.142, respectively; p < 0.001 and p = 0.003, respectively) (Table 2). However, none of these interactions was explained by small or large fish systematically leading in the pair with respect to speed and turn rate (time-lag between small and large fish: −0.025 and 0.022 s, respectively; p = 0.052 and p = 0.096, respectively) (Table 2).

### Shoaling and Schooling are affected by Behavioral Teleporting

Additional analyses on the qualitative interaction between the animals indicate that extent of schooling and shoaling tendency between subjects was reduced by behavioral teleporting (Text S2 and Figure S4). These reductions did not affect the ability of the approach to preserve natural interactions, whereby Control and Match subjects aligned their motion when in close proximity of a conspecific (Control) or a replica (Match), and such a relationship was not observed in the Mismatch condition (Text S2 and Figure S5).

## Discussion

In this paper, we designed and validated a robotics-based approach to perform hypothesis-driven experiments on zebrafish social behavior. Through the notion of behavioral teleporting, our approach enables fine control over locomotory patterns and morphological features of robotic stimuli. We demonstrated the possibility of transferring the behavior of a live animal onto a remotely-located robotic replica that consistently interacted with another live animal. Through parallel behavioral teleporting, we explored the interaction between two live zebrafish swimming in remotely-located tanks: each fish interacted with an inanimate robot which mirrored the locomotory patterns of the other subject.

Across all trials, we demonstrated the possibility of swiftly teleporting the behavior of live animals onto robotic replicas with a high degree of precision with respect to position, speed, turnrate, and acceleration. We believe that the efficacy of our platform was instrumental to the success of the present study. Previous endeavors in the field of interactive fish-robot interactions (Bonnet et al., 2016; Cazenille et al., 2018; De Lellis et al., 2020; Kim et al., 2018; Kopman et al., 2013; Landgraf et al., 2013, 2016; Papaspyros et al., 2019) have documented unexpected fish reactions to a robotic stimulus inspired by a conspecific. Our platform, instead, elicited the responses that should be predicted on the basis of fish-to-fish social behavior. This improved level of biomimicry should be attributed to the theoretical twist of this study, which embraces behavioral teleporting rather than mathematical representations of fish behavior. By obviating the need of a mathematical model to control the robotic stimulus, our methodology is devoid of any approximation that could skew the appraisal of the stimulus by the live animal. Our approach to maneuver the robotic replica does not rely on classical behavioral rules inspired by schooling and shoaling (Bonnet et al., 2016; Kim et al., 2018; Kopman et al., 2013; Landgraf et al., 2013, 2016), or on recent data-driven stochastic models of fish behavior (De Lellis et al., 2020; Papaspyros et al., 2019), or on probability-based feedback mechanisms (Cazenille et al., 2018).

The interaction between live animals swimming in the same or in different tanks was studied through the lens of information theory (Bossomaier et al., 2016). Specifically, we adopted the concept of transfer entropy (Schreiber, 2000) to quantify the extent to which the knowledge about the past behavior of one fish could reduce the uncertainty in predicting the behavior of the other fish from its past behavior. To increase statistical power, we opted for a symbolic representation (Staniek and Lehnertz, 2008) of the time-series of animal locomotion, where each symbol encoded a specific locomotory bout of the fish ethogram. In agreement with our expectations, we registered a strong mutual influence between the subjects in all experimental conditions (Control, Match, and Mismatch conditions). This finding demonstrates the feasibility of behavioral teleporting onto a replica to allow interaction between two remotely-located fish.

Upon such a robotics-mediated interaction, it is possible to devise hypothesis-driven experiments in which locomotory patterns must be faithfully reproduced by an inanimate robot and potentially dissociated from other intervening variables. The present experiments on the role of morphology in social behavior offer compelling evidence in favor of this proposition. Although coupling morphology with locomotory patterns during behavioral teleporting (Match condition) resulted in the preservation of the natural interaction between two live animals (Control condition), introducing a conflict between morphology and locomotory patterns (Mismatch condition) begot unnatural interactions. These manipulations are unprecedented in the field of animal-robot interactions, but they have roots in other fields of animal behavior. Extremely common in nature are instances of evolutionary-driven deceptions, in which an animal would change its behavior and appearance to mimic other conspecifics toward gaining some advantage. For example, in sexual mimicry, sneaky copulation is often pursued by small male fish that will mimic females to gain access to their female territory and enhance chances of copulation with them (Hanlon et al., 2005; Norman et al., 1999). Likewise, aggressive or defensive mimicry represents other instances of animals adopting traits that are not typical for their sex and age to improve their fitness (Randall, 2005).

The interaction between control subjects, which is preserved by behavioral teleporting in the Match condition, points at an asymmetric relationship between fish of different size. Specifically, transfer entropy from the small to the large fish is larger than transfer entropy from the large to the small fish, thereby denoting the small fish as the one driving the interaction. Delving into the qualitative nature of the interaction through cross-correlation analysis, we observed that the stronger influence of the small fish was explained by a tendency to initiate new maneuvers that were followed by the large fish. It is tenable that such an interaction could represent an instance of leader-follower relationship (Krause et al., 2000). The possibility of small fish leading larger individuals has already been documented in prior work on freshwater fish (Reebs, 2001), although conclusive evidence in favor of an association between leadership and body size is yet to be elucidated (Harris et al., 2010; Reebs, 2001; Ward et al., 2002).

One of the key findings of our study is that, in contrast with the Match condition, Mismatch subjects failed to replicate the large-to-small fish relationship observed in Control subjects. In the Mismatch condition, live fish swam together with replicas of their same size; yet, while the large replica replicated the behavior of the small fish, the small replica replicated the behavior of the large fish. Within this setting, we failed to observe the emergence of a robotics-mediated asymmetric interaction between the remotely-located fish. Additionally, animals did not display the natural tendency of aligning their swimming directions when they were in close proximity (Miller and Gerlai, 2012), which was registered in Control and Match conditions. Hence, any disruption in the association between locomotion and morphology can result in the lack of natural fish-to-fish relationships. This evidence strengthens the view that social dynamics depends on both locomotory patterns and morphological features, which could underlie the origins of leadership (King et al., 2009).

The approach presented herein opens several research avenues in preclinical models of physiology and disease. One of the main hurdles in preclinical research relates to the fact that behavior is inextricably linked to underlying endophenotypes, such as hormones, circadian rhythms, and gene expression. Hence, model subjects could present alterations that exceed the behavioral ones they were meant to present. For example, genetically modified or pharmacologically manipulated zebrafish may exhibit aberrant endophenotypes that could challenge fine control of experimental variables in the study of social behavior (Grossman et al., 2010; Kim et al., 2017). Ethanol may represent an epitome: its administration in zebrafish results in both behavioral (locomotion) (Ladu et al., 2014) and morphological (coloration) (Gerlai et al., 2000) alterations. Under these conditions, discriminating whether the influence of ethanol on sociality is mediated by behavior or morphology becomes impossible. Behavioral teleporting can help addressing this issue.

### Limitations of the Study

In spite of anticipated advantages, our approach presents, nonetheless, several limitations. First and foremost, the locomotion of the replicas should be improved to reflect the complex behavioral repertoire of zebrafish in three dimensions. In the present study, to demonstrate the feasibility of our approach, experimental subjects (and replicas) were constrained to shallow water, thereby preventing the onset of complex species-specific three-dimensional trajectories (Kalueff et al., 2013). Although this decision was functional to the scopes of our study, we acknowledge that future incarnations shall contemplate three-dimensional behavioral teleporting and scoring. This main hurdle depends on both the computational load necessary to reconstruct three-dimensional trajectories of live fish and, most importantly, hardware requirements to design untethered robotic stimuli that could autonomously swim along three-dimensional trajectories.

Another key aspect to be improved is in the extent of biological mimicry. Although the replicas we devised allowed preserving several aspects of the interactions between live animals swimming in the same tank, they nonetheless failed to maintain the same absolute values of shoaling and schooling tendencies registered in Control subjects. A number of factors might contribute to this limitation, including incomplete replication of body undulations through mere passive compliance, imperfect pigmentation and stripe patterning, lack of moving fins and gills, absence of olfactory cues similar to live animals, and undesired mechanical vibrations from the robotic platform. All these factors are in fact known to contribute to the appraisal of other fish by zebrafish individuals (Engeszer et al., 2004, 2008; Polverino et al., 2012; Rosenthal and Ryan, 2005; Saverino and Gerlai, 2008; Yoshihara, 2014).

Another potential limitation of this study relates to the selection of control subjects. Specifically, resting upon the objective to investigate the effect of differential body size, we conducted the study on subjects characterized by major differences in body length. This resulted in a fish population consisting of large and small individuals. Having two “extreme” populations did not allow resolution of the absolute effect of body size, whereby we lacked a subject of intermediate size that could represent a reference comparison. Future studies could involve a larger fish population, in which one could examine robotics-mediated interaction between remotely-located live animals of small and intermediate sizes as well as animals of large and intermediate sizes.

### Resource Availability

#### Lead Contact

Further information and requests for resources should be directed to and will be fulfilled by the Lead Contact, Maurizio Porfiri (mporfiri@nyu.edu).

#### Materials Availability

This study did not generate any materials.

#### Data and Code Availability

Datasets and MATLAB scripts can be downloaded from the GitHub repository of the Dynamical Systems Laboratory at New York University: https://github.com/dynamicalsystemslaboratory/BehavioralTeleporting.

## Methods

All methods can be found in the accompanying Transparent Methods supplemental file.

## Supplemental Information

Document S1. Transparent Methods, Figures S1–S7, Table S1, and Text S1–S4

Figure360. An Author Presentation of Figure 1
