# Supplementary material for: Behavioral Teleporting of Individual Ethograms onto Inanimate Robots: Experiments on Social Interactions in Live Zebrafish
Source: iScience. 2020 Jul 29;23(8):101418. doi: 10.1016/j.isci.2020.101418 (PMC7452384; doi:10.1016/j.isci.2020.101418)
Supplement: Document S1. Transparent Methods, Figures S1–S7, Table S1, and Text S1–S4 [file mmc1.pdf]

iScience, Volume *23*

Supplemental Information

Behavioral Teleporting of Individual Ethograms onto Inanimate Robots: Experiments on Social Interactions in Live Zebrafish

Mert Karakaya, Simone Macrì, and Maurizio Porfiri

Supplemental information

Text S1. Performance of the robotic platform, related to Figure 2

In addition to the similarity index considered in the main document, we examined the performance of the platform by comparing the speed, turn rate, and magnitude of the acceleration of the replica to a live fish. Specifically, we conducted a cross-correlation analysis, equivalent to the one explained in the main document, for all of these metrics. We limited the analysis to the 20-s time-intervals that were deemed successful in terms of the similarity index. For each interval, we computed the maximum value of the normalized cross-correlation and the corresponding time-lag. Different from the main document, we extended the range of admissible time-lags to 0.4 s, to acknowledge the numerical differentiation required in the calculation of speed, acceleration, and turn rate. We calculated a total of 1018 values of the maximum cross-correlation values for speed, turn rate, and magnitude of the acceleration with averages (standard errors) of 0.919 (± 0.002), 0.730 (± 0.003), and 0.849 (± 0.002), respectively (Fig. S1).

Text S2. Additional spatio-temporal analysis of live fish behavior, related to Figure 3 and Table 1

To investigate the behavioral similarity among the three experimental groups, we assessed speed, turn rate, and the magnitude of the acceleration in large and small fish tested in Control, Match, and Mismatch conditions. These data were analyzed through repeated measures ANOVA for split-plot designs with one between-subject factor (size: large versus small) and two within-subject factors (condition: Control versus Match versus Mismatch; time-bins: five 2-min intervals). For Match and Mismatch conditions, we limited the analysis to the 20-s time-intervals that were deemed to be successful by the similarity index described in the main document.

From our analysis, we conclude that speed was comparable between large and small individuals ($F_{1,18}$=1.68, p=0.212; Fig. S2A,B). While experimental groups apparently swam at a different speed ($F_{2,36}$=3.54, p=0.039; Fig. S2A,B), post-hoc comparisons failed to identify significant pairwise differences between conditions. Likewise, we recorded a variation of the speed over time ($F_{4,36}$=4.61, p=0.002; Fig. S2A,B); post-hoc analyses revealed that speed was higher between minutes 3-6 than 9-10 ($p<0.05$; Fig. S2A,B). With respect to turn rate, neither did we register a difference with respect to the size of the animals ($F_{1,18}$=0.68, p=0.420; Fig. S2C,D), nor with respect to the experimental condition ($F_{2,36}$=0.60, p=0.555; Fig. S2C,D). The magnitude of the turn rate changed over time ($F_{4,36}$=32.03, $p<0.001$; Fig. S2C,D), with post-hoc pairwise comparisons indicating a decline from minutes 1-2 to 9-10 ($p<0.05$; Fig. S2C,D). The analysis of the acceleration magnitude paralleled that of the speed, whereby we did not register an effect of size ($F_{1,18}$=1.51, p=0.235; Fig. S2E,F) and differences in conditions ($F_{2,36}$=4.75, p=0.015; Fig. S2E,F) did not translate into significant pairwise comparisons. The acceleration magnitude varied in time ($F_{4,36}$=16.54, $p<0.001$; Fig. S2E,F), all animals exhibiting higher acceleration during the early stage of the experimental session than towards the end ($p<0.05$; Fig. S2E,F). Alongside with analysis of general locomotion, we examined the spatial distribution of all the animals across experimental conditions, confirming equivalent response of each animal when tested with another conspecific or a replica through behavioral teleporting (Fig. S3).

In addition to examining temporal and spatial patterning of swimming activity, we analyzed the extent to which fish pairs coordinated their motion across experimental conditions in terms of schooling and shoaling (Miller and Gerlai, 2012; Tunstrøm et al., 2013). The analysis of robotics-mediated interactions of remotely-located fish in Match and Mismatch conditions was performed by using local reference frames in each tank. In practical terms, the trajectories of the fish in the two different tanks were superimposed on the same reference frame. Similar to the main text and the analysis presented above, for Match and Mismatch conditions, we only considered the 20-s time-intervals in which the platform performed as expected. We used a repeated measures ANOVA with two within-subject factors (condition: Control versus Match versus Mismatch; time-bins: five 2-min intervals). Tukey's honestly significant different (HSD) post-hoc tests were used when allowed. Statistical significance was set at 5%.

To quantify schooling tendency, we utilized polarization (Miller and Gerlai, 2012; Tunstrøm et al., 2013), which measures the extent to which fish aligned their orientation (Fig. S4A). Polarization varies between zero and one, where 1 indicates fully coordinated swimming with both fish swimming in the same direction. Highly coordinated swimming was observed in the Control condition, with an average polarization of 0.725. In the Match and Mismatch conditions, average polarizations of 0.635 and 0.640 were observed, respectively. Such a difference between Control individuals and subjects in Match and Mismatch conditions was confirmed by statistical analysis ($F_{2,18}$=25.67, p<0.001; p<0.05 in post-hoc tests; Fig. S4A) and was consistent over time ($F_{4,36}$=0.07, p=0.991; Fig. S4A).

To score shoaling tendency, we measured the distance between the live fish (Miller and Gerlai, 2012; Tunstrøm et al., 2013) (Fig. S4B). Control fish maintained an average distance of 7.098 cm. Average distances of 17.964 cm and 18.759 cm were registered for Match and Mismatch Chance pairs, respectively. Similar to the analysis of polarization, we determined that the distance between Control fish was smaller than the distance between Match and Mismatch pairs ($F_{2,18}$=48.96, p<0.001; p<0.05 in post-hoc tests; Fig. S4B) and that this effect was consistent over time ($F_{4,36}$=0.32, p=0.866; Fig. S4B).

Complementing the analysis of the temporal patterning of fish interactions, we assessed the extent to which behavioral teleporting maps the behavior of Control pairs onto the behavior of Match and Mismatch pairs. Such an assessment was performed through a further correlation analysis between schooling and shoaling. Once again, we focused on the time-intervals of Match and Mismatch conditions in which behavioral teleporting was successful (as determined in the main document). Chance values were calculated using fish from different trials of the Control condition, so that the Chance group consisted of 12 fish pairs of one small and one large animal, randomly selected from the Control condition. We utilized Matlab R2019b Statistics Toolbox and used the fitlm function to compute R^2 and the p-values. Predictably, schooling and shoaling patterns of Control pairs were significantly correlated (R^2=0.361, p<0.001; Fig. S5A), so that fish would tend to swim along the same direction when in close proximity. This association was preserved in the Match condition (R^2=0.044, p<0.001; Fig. S5B), but it was lost in the Mismatch condition (R^2=0.011, p=0.111; Fig. S5C). Further, the analysis on the values obtained by chance showed that this dependency does not exist between two random fish (R^2=0.006, p=0.161; Fig. S5D).

Overall, these findings suggest that live fish did not perceive the replicas as an attractive stimulus in comparison with other conspecifics. A possible explanation for this claim is that all the experimental fish were naïve to the replicas, whereby they were introduced to the stimulus for the first time during the trials, different from Control subjects that habituated to each other for two days in the same housing tank. It is tenable that 10 min of experimental time might not be sufficient to acclimate to a robotic stimulus. At the same time, behavioral teleporting was successful in selectively preserving the association between shoaling and schooling, whereby Match pairs were found to align their motion when swimming in close proximity to the replicas, similar to Control subjects swimming in the same tank.

Text S3. Fish body size, related to Figure 1

The selection of fish (small versus large) for the experiments was based on the measurement of their body size, which was conducted as follows. First, we gently hand-netted the fish from their collective housing tanks into a shallow plastic beaker filled with water. Then, we placed the beaker on top of a grid paper and took a high-resolution photo of the fish with an overhead camera (iPhone X, Apple, Cupertino, CA, USA). Once the photo was taken, the fish was placed back in the holding tank for the experiments. Using an open-source software (ImageJ, National Institute of Health, Bethesda, MD, USA), we measured the body length of the fish with respect to the millimeter scale of the grid paper. We obtained an average body length of 25.9 mm (25.2 mm) and 31.8 mm (36.0 mm) for small and large males (females), respectively. Across all pairs, large fish were 32.7% larger than small fish. Individual measurements of all the fish used in this study are reported in Table S1.

Text S4. Parametric study on transfer entropy, related to Table 1

Toward the rigorous application of transfer entropy to examine fish interaction, we conducted a parametric study to determine the optimal resolution that minimizes noise. Following standard practice (Bossomaier et

al., 2016), we computed entropy as a function of the sampling time ranging from 0.1 s to 1 s with a resolution of 0.05 s, and we chose the resolution at which entropy was minimized. The optimization was carried out on the symbolic time-series of all the animals across all conditions (a total of 72 time-series). The initial value of the sampling-time was based on the software and hardware constraints of the robotic platform, whereby the use of physically unrealistic sampling-times might beget false positive results (Weber et al., 2017). Results from the analysis indicate an optimal resolution of 0.1 s (Fig. S6A).

For the Control condition, interaction between two fish was assumed to be instantaneous between consecutive time-steps (Butail et al., 2016; Collignon et al., 2019; Strandburg-Peshkin et al., 2013). For the Match and Mismatch conditions instead, one should expect a time-delay in the interaction between the remotely-located animals. To determine such a delay, we evaluated the sum of the transfer entropy values from the small to the large fish and from the large to the small fish in the Match condition, for different time-delays varying from 0 to 4 time-steps (0 s to 0.4 s). The sum of the transfer entropy values is indicative of the overall strength of the interaction between the animals (Wibral et al., 2013). Although the dependence of the sum of the transfer entropies on the time-delay was minimal, a local peak corresponding to 0.2 s can be identified (Fig. S6B). This value of the time-delay was in agreement with our predictions, whereby we estimated a total time of 0.2 s for the simultaneous behavioral teleporting across the tanks. Hence, we used a delay of 0.2 s when scoring behavioral interactions in the Match and Mismatch conditions, with respect to both transfer entropy and cross-correlation analyses.

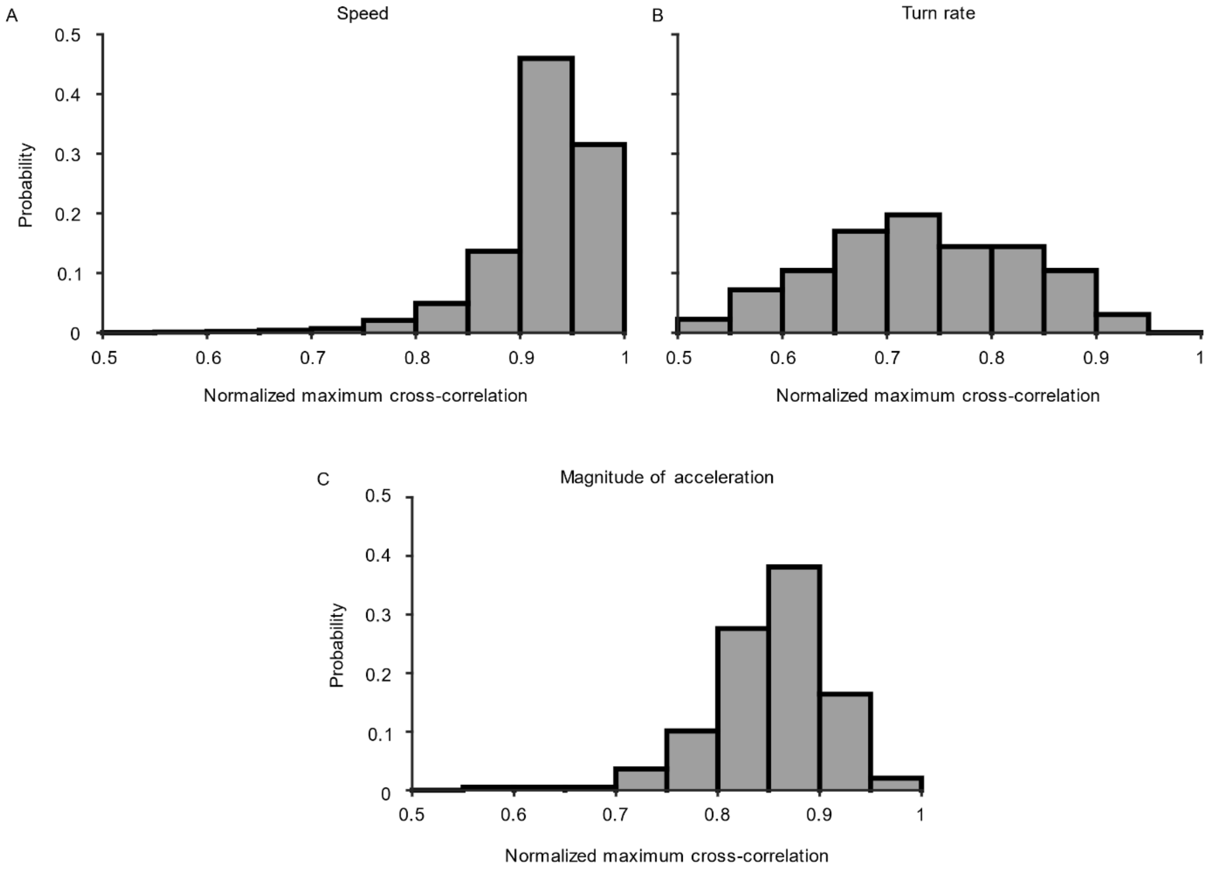

Figure S1. Additional performance analysis of the robotic platform, related to Figure 2. Histograms of normalized maximum cross-correlation between the robotic replica and the corresponding fish whose behavior is being teleported for all the acceptable trials. The analysis is conducted for three different metrics: **A**) speed, **B**) turn rate, and **C**) magnitude of the acceleration.

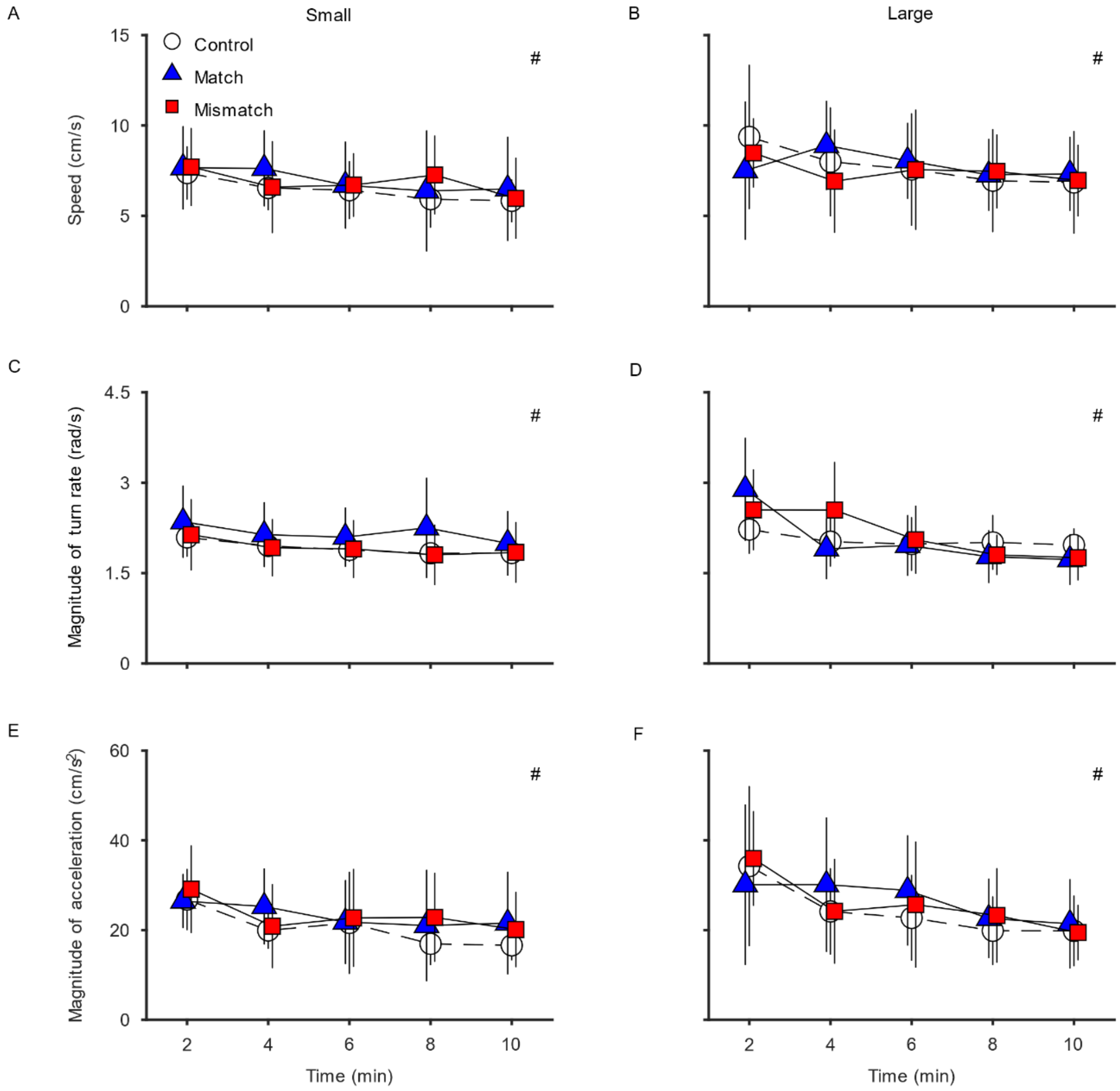

Figure S2. Temporal patterning of swimming activity of live fish, related to Figure 3 and Table 1. The analysis is conducted for: **A,B**) speed, **C,D**) magnitude of the turn rate, and **E,F**) magnitude of the acceleration, for **A,C,E**) small and **B,D,F**) large fish. White circles refer to Control condition, blue triangles to Match condition, and red squares to Mismatch condition. Data are expressed as means ± standard deviations. # indicates a significant time-effect at 5% significance level in post-hoc comparisons.

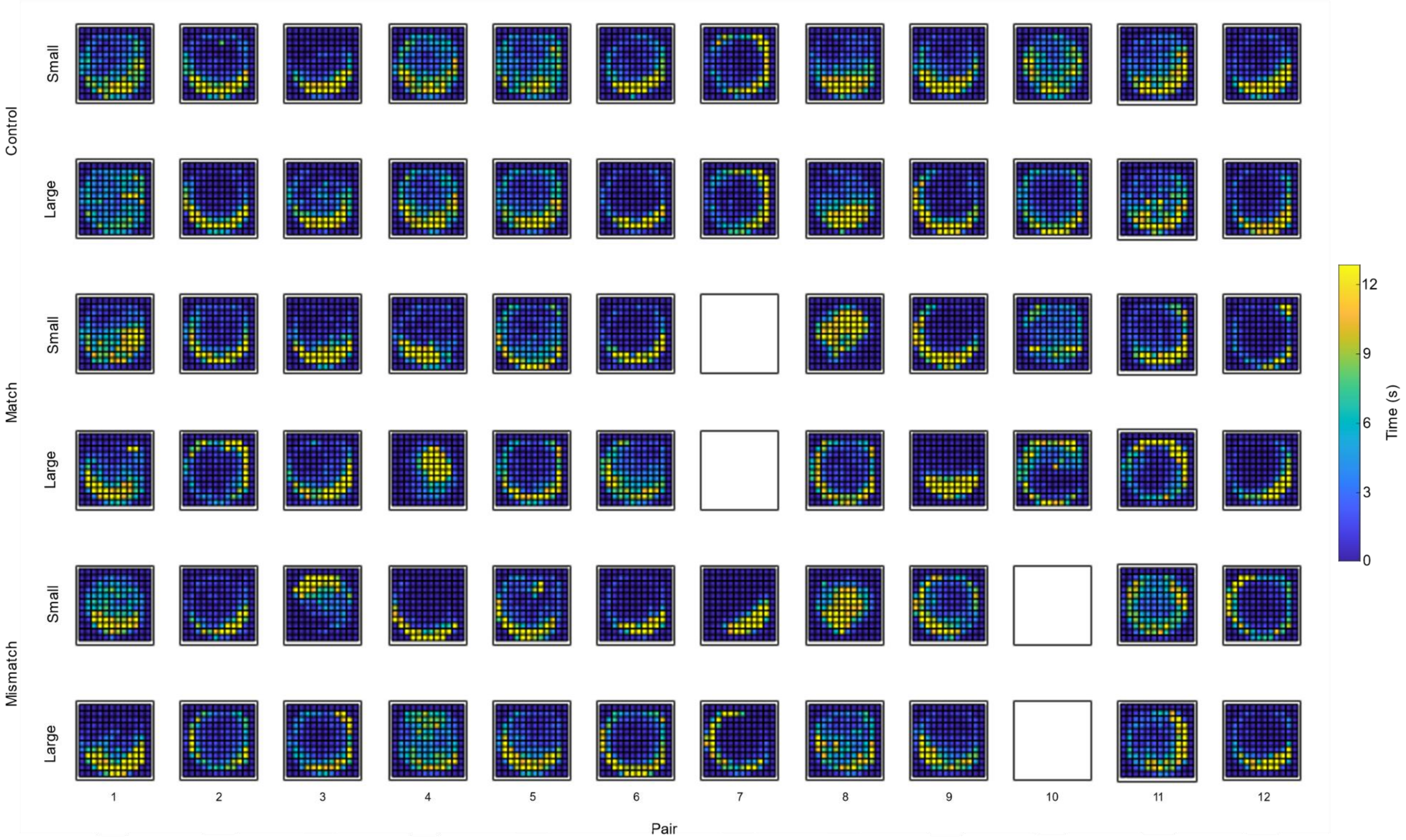

Figure S3. Spatial distribution across all experimental trials, related to Figure 3 and Table 1. The color indicates the time by each live fish in different portion of the experimental tank. Empty frames correspond to trials that were excluded from the analysis due to technical issue with the robotic platforms.

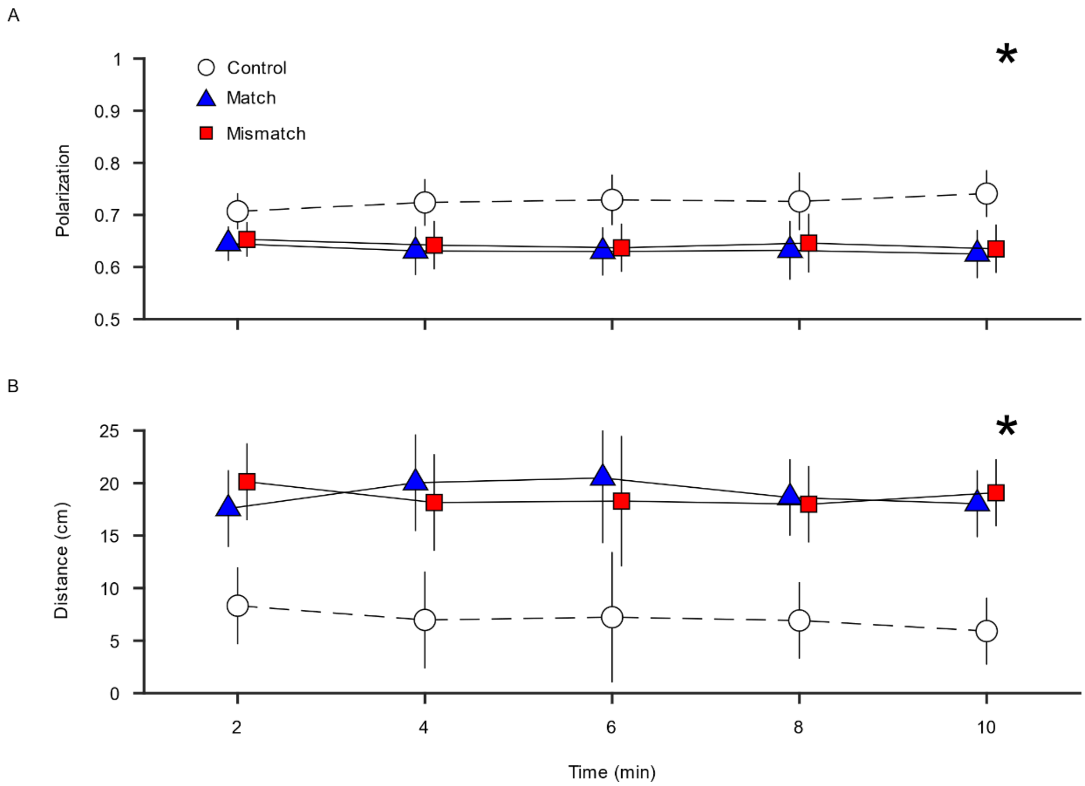

Figure S4. Temporal patterning of the interaction between two live animals, related to Figure 3 and Table 1. A) Polarization of the two live fish in a given pair, as a function of time and condition. **B**) Distance between two live fish in a given pair, as a function of time and condition. White circles refer to Control condition, blue triangles to Match condition, and red squares to Mismatch condition. Data are expressed as estimated marginal means ± standard deviations. * indicates $p<0.05$ in post-hoc comparisons between Match/Mismatch conditions and Control.

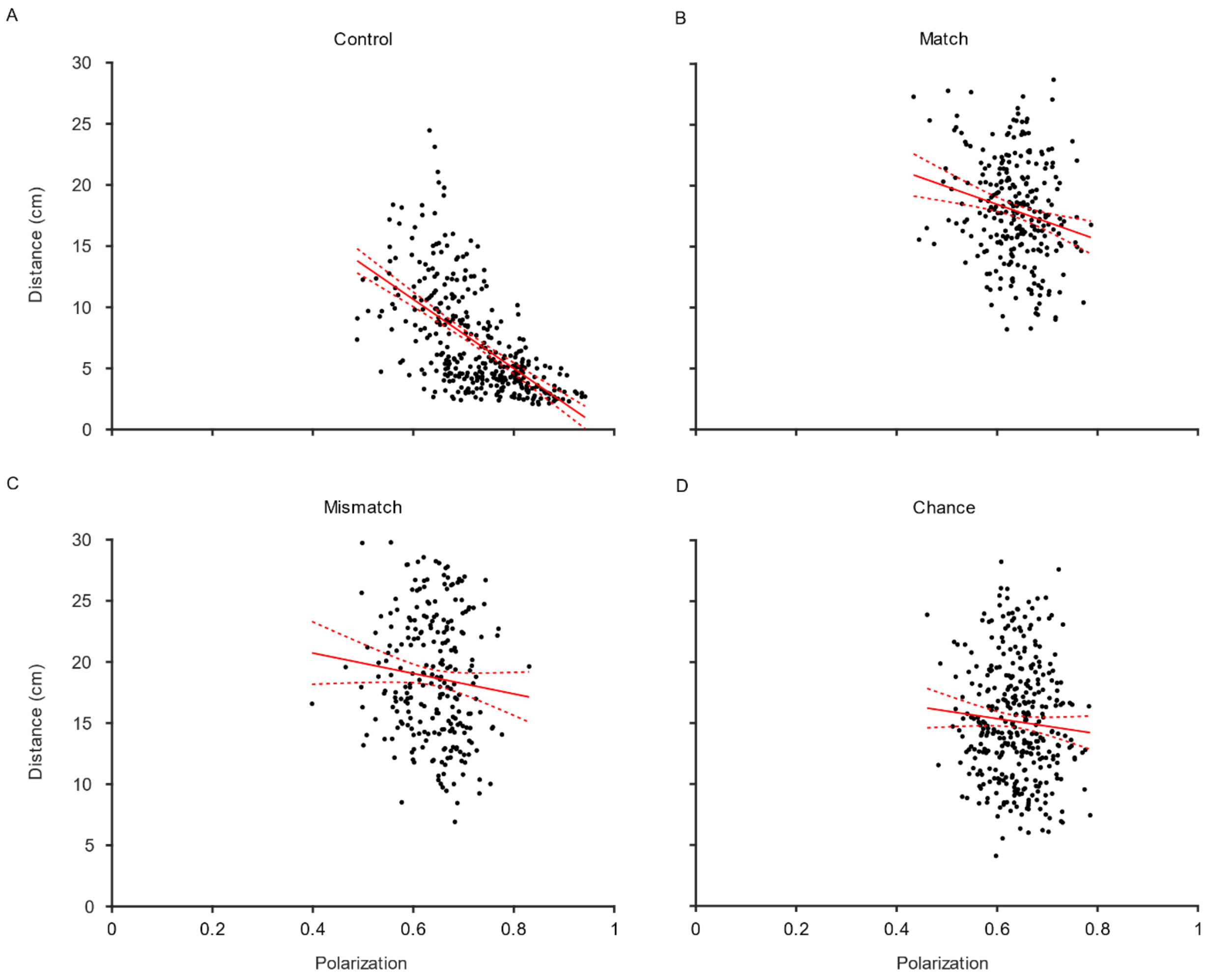

Figure S5. Correlation between shoaling and schooling, related to Figure 3 and Table 1. Linear regression analyses between the distance between two fish and their polarization. **A**) Control; **B**) Match; **C**) Mismatch; and **D**) Chance pairs. For the case of Match and Mismatch pairs, the calculation is limited to the time-intervals deemed successful in the similarity index analysis in. Black points are individual data points, solid red line is the linear regression line, and dashed red lines are the confidence intervals.

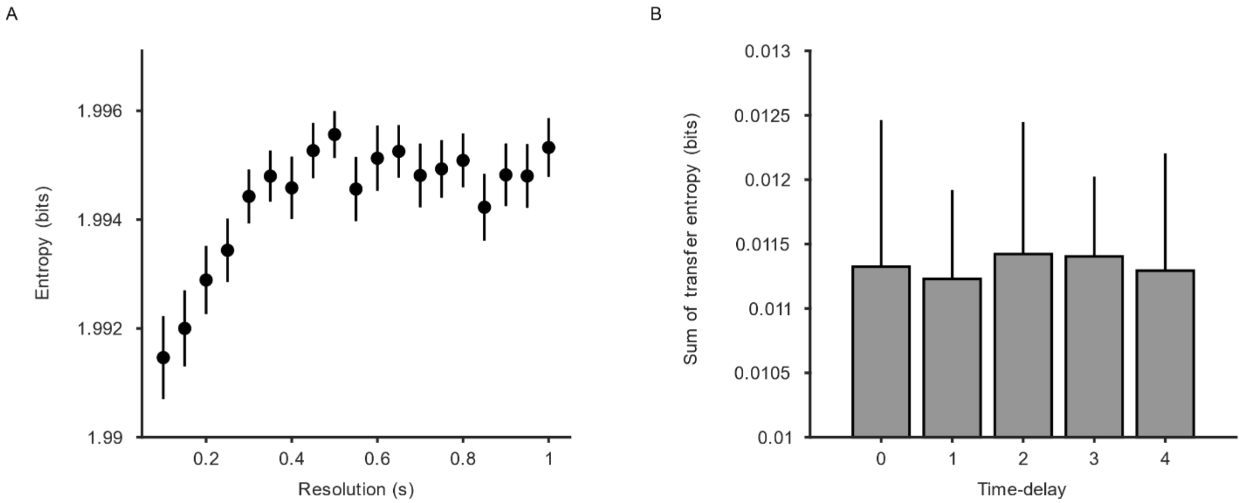

Figure S6. Parametric analysis for transfer entropy, related to Table 1. A) Downsampling analysis on the entropy of all the subjects to select the optimal resolution for transfer entropy computations; black dots are entropy values for a given resolution and lines are standard errors. **B**) Time-delay analysis for the Match condition to estimate the physical delay due to behavioral teleporting; lines represent standard error.

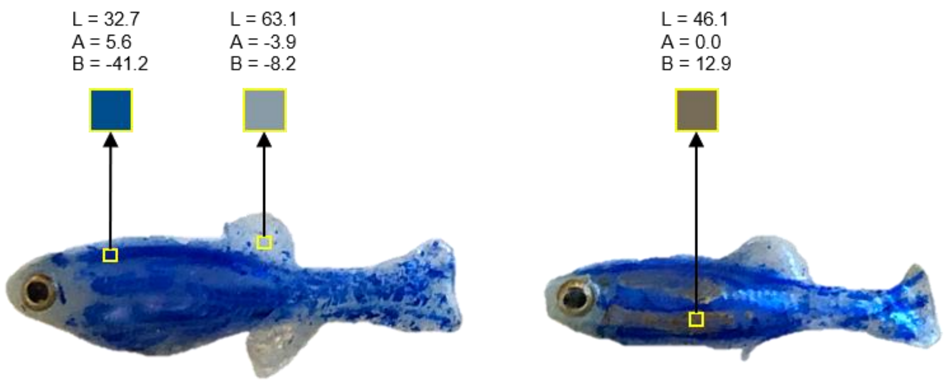

Figure S7. Color measurements of the replicas in CIELAB space from spectral analysis, related to Figure 1. The color of the stripe pattern of the replicas is shown in the left and right measurements, while the color of the silicone used for the mold is shown in the middle measurement. Left is a replica of a female subject and right is the replica of a male subject.

Pair	Large (mm)	Small (mm)
M-1	35.5	27.0
F-1	35.6	26.5
M-2	33.2	29.5
F-2	35.6	26.2
F-3	28.7	26.0
M-3	35.0	23.7
M-4	30.8	24.4
F-4	38.8	24.5
M-5	32.2	26.0
F-5	34.6	25.3
F-6	30.2	23.0
M-6	36.3	25.2

Table S1. Fish body size, related to Figure 1. Body size of all the experimental subjects employed in the study. “M” and “F” identify whether the pair is composed of male or female subjects, and the adjacent number labels the pair.

	Day 1		Day 2		Day 3	
Morning	M-1	Control	M-1	Match	M-1	Mismatch
	F-1	Control	F-1	Match	F-1	Mismatch
	M-2	Match	M-2	Mismatch	M-2	Control
	F-2	Match	F-2	Mismatch	F-2	Control
	F-3	Mismatch	F-3	Control	F-3	Match
	M-3	Mismatch	M-3	Control	M-3	Match
Afternoon	M-4	Control	M-4	Mismatch	M-4	Match
	F-4	Control	F-4	Mismatch	F-4	Match
	M-5	Match	M-5	Control	M-5	Mismatch
	F-5	Match	F-5	Control	F-5	Mismatch
	F-6	Mismatch	F-6	Match	F-6	Control
	M-6	Mismatch	M-6	Match	M-6	Control

Table S2. Counterbalancing procedure, related to Figure 1. Allocation, order of testing, and timing of experimental pairs to the three different conditions.

Transparent methods

Experimental setup

The experimental apparatus consisted of two cylindrical tanks on an elevated base, two robotic platforms, two webcams, and a desktop computer. The elevated base, made of aluminum T-slot bars (McMaster-Carr, Elmhurst IL) and a 6.3 mm thick plywood panel (60 cm × 120 cm, width × height; Home Depot, Atlanta GA), was placed at the center of the experimental area (Fig. 1A). Both manipulators were positioned side-by-side under the base. Two circular tanks (36 cm × 15 cm, diameter × depth) were placed on the base, centered with respect to the manipulators. Green tanks were chosen to confer a homogenous background for tracking and offer a less stressful environment for fish (Oliveira et al., 2015). Two 250 lumen LED lamps (Target, Minneapolis MN) were positioned 75 cm above the base to provide a uniform white background (ambient lighting: 200 lux). Two overhead cameras (Logitech C920 Pro, Logitech, Lausanne, Switzerland) were mounted 75 cm above the tanks to record the experiments, at a resolution of 640 × 360 pixels and an acquisition rate of 20 frames per second. The cameras and platforms were connected to an HP Z4-G4 workstation (HP, Palo Alto CA), which served the twofold purpose of: i) performing real-time tracking of live fish and replicas in both tanks, and ii) maneuvering each of the replicas to mirror the motion of the fish in the other tank.

Robotic platform and live tracking system

In order to maneuver the replicas along biologically relevant locomotory patterns through nonintrusive actuation, we used a custom-designed robotic manipulator (Fig. 1A). This device consisted of a two-dimensional Cartesian manipulator with two NEMA 17 stepper motors along each axis (MakeBlock XY Plotter, MakeBlock, Shenzhen China). The workspace of the Cartesian manipulator was 390 mm by 320 mm (length × width). A magnetic system was used to couple the end-effector with the replica, similar to other experimental setups in the literature (Bonnet et al., 2016; Landgraf et al., 2016; Romano et al., 2020). Specifically, a neodymium magnet (25.4 mm × 6.4 mm, diameter × thickness; McMaster-Carr, Elmhurst IL) was placed on a 3D-printed end-effector, which was tethered to the manipulator (Fig. 1A). Another magnet (6.4 mm × 6.4 mm, diameter × thickness; McMaster-Carr, Elmhurst IL) of opposite polarity was housed inside the replica such that it would be pulled along the paths traversed by the manipulator, without physical contact and at a minimal friction. Different from De Lellis et al. (2020), we did not include a dedicated motor for rotating the replica to reduce computational burden and mechanical noise in the tank.

The manipulator was driven by a microcontroller (Arduino Uno, Arduino, Italy), interfaced with a stepper motor shield (Kuman CNC Shield, Kuman, China) for signal generation/modulation and power supply. Closed-loop control was implemented through the Grbl(tm) v0.9 library (Jeon, 2011), a software for stepper motor control in Arduino. Use of stepper motors and low friction rails allowed an accurate position and velocity control without feedback, guaranteeing a resolution of 0.2 mm and a smooth motion of the end effector with minimum hysteresis.

We developed a tracking software to identify fish and replicas with high accuracy, while distinguishing their identities throughout the trial. Before each experimental session, the tracking software was initialized. During this phase, the robotic platform was calibrated using a homing sequence, where the end-effector was positioned at the center of the working space. Kalman predictors were reset and video writers were set-up. Next, the replica was magnetically connected to the end-effector.

Following the initialization phase, we cropped the video streams from the webcams into 400 × 400 pixel size, centering the experimental tanks in the images. From these cropped images, we manually identified a circular region of interest over the experimental area, and created a binary mask based on the selected region. The binary image frame aided in eliminating uncertainty due to external factors, such as lighting and shadows. To facilitate identification of the targets, we duplicated the masked frames and changed the image space of the duplicated image from red-green-blue (RGB) to hue-saturation-value (HSV). By applying image thresholds, we created two binary masks, which together served to identify both targets and keep their identities. From the saturation and value layers, we identified the two targets against the homogeneously illuminated background in the experimental region. To resolve the identity of the replica

from the fish, we used the hue layer, from which we identified the blue pigment of the silicone replica (not present in the live fish).

Toward ultimately determining the location of the two targets, we used a blob analyzer in Matlab R2019b (Mathworks MA, USA). The blob analyzer recognized connected pixels in the binary masks, from which we inferred the location of a target as the centroid of the corresponding blob. To improve on the resolution of the identities during potential occlusions, we filtered instances in which the fish centroid was closer than 20 pixels to the replica centroid. A Kalman predictor was then used to solve these instances. The predictor used the position of the two targets prior to the potential occlusion, assuming their velocities were constant (Blackman, 1986). The confidence values between the Kalman predictions and all the detected objects were calculated to generate a cost matrix, which was employed to identify the most probable object locations through Munkres' assignment algorithm (Munkres, 1957).

Once the centroid positions were identified, the position of each fish was transferred to the replica in the opposite tank, thereby affording behavioral teleporting of fish motion. The overall delay in transferring fish motion to the replica was approximately 40 ms: 10 ms was the time required to analyze images in each tank and 30 ms was the communication cycle of each microcontroller. Therefore, to minimize delay and interference of data transfer between the microcontrollers, we updated the positions of the replicas every 100 ms. We minimized confounds associated with fish tracking uncertainty, by averaging the position of the fish in the previous and current frames before sending the command to the microcontrollers. Due to physical limitations of the stepper motors, the maximum speed of the replicas was set at 20 cm/s, so that a single position update would consist of a motion of at most 2 cm.

Replica

To investigate the interaction between robots and live fish, we designed a conspecific-like replica based on a live zebrafish (*Danio rerio*) (De Lellis et al., 2020). We tested four different replicas, whose dimensions were based on the average size of experimental fish: a small male (26 mm in length), large male (32 mm in length), small female (25 mm in length), and large female (36 mm in length) (Fig. 1B) (Supplemental Information: Text S3 and Table S1).

The replicas were created in skin-safe silicone (Dragon Skin 10, Smooth – On, USA), toward a soft body capable of undulating during maneuvers, similar to previous studies (Bonnet et al., 2016; De Lellis et al., 2020; Romano et al., 2019). The original three-dimensional computer-aided-design (CAD) of a zebrafish developed by Kim et al. (2018) was used as a reference to create the four replicas. Specifically, by tailoring the morphology of the original model according to acquired pictures of live animals, we 3D-printed four rigid models in polylactic acid (PLA) filament using a MakerBot Replicator 5 (MakerBot, New York, NY, USA). Then, the molds for the four silicone replicas were produced from polyethylene terephthalate glycol (PETG) using a vacuum forming machine over the 3D-printed models. Molds were filled with skin-safe silicone (Dragon Skin 10, Smooth – On, USA) to create replicas, which were painted with non-toxic acrylic paint (Smooth – On, USA).

We painted the replicas using silicone pigments and silver specks, following natural coloration patterns (males in gold and blue patterns, and females in silver and blue patterns). The coloration patterns of the replicas in CIELAB color space were measured using a spectrometer (USB2000, Ocean Optics, FL, USA), similar to (Romano et al., 2020) (Supplemental Information: Fig. S7). To improve the realism of replicas, two glass eyes (Van Dyke Supply Co., Granite Quarry, NC, USA) were glued onto the replicas (Landgraf et al., 2016). Finally, a 0.75 mm diameter wire was inserted through the middle of the replica to create a spine that would provide a mechanical connection to the magnetic base.

The base to which the replica was tethered comprised a plastic shaft and a magnetic attachment. The plastic shaft consisted of a 4 cm hollow conical body, through which the wire extending from the replica spine was inserted. The wire was free to rotate around its axis in the shaft, allowing the replica to passively align itself with the swimming direction. The plastic shaft was glued to a 3D-printed support, housing the magnet.

Experimental procedure

Adult wild-type zebrafish were purchased from Carolina Biological Supply (Burlington NC, USA). Upon delivery, all animals were allowed for an acclimation period of at least 14 days to the laboratory conditions. Photos of individual fish were taken on a millimeter paper background to measure their body length. We formed pairs of the same sex, with a small and a large fish. Each pair was housed in a separate tank for a two-day habituation period (Supplemental Information: Table S1), and each pair was tested in all three conditions in a counterbalanced order over three consecutive days (Supplemental Information: Table S2). All animals were kept under a 12 hour light-dark cycle, with lights on at 9:00 AM at an intensity range between 50 and 100 Lux. Water temperature was maintained at 26ºC. In total, we tested 12 male and 12 female fish.

Before each of the two daily experimental sessions (morning and afternoon), the tanks were washed and filled with 26ºC water up to 6 cm in depth. Six male and six female pairs were tested for three conditions, namely: “Control,” “Match,” and “Mismatch” (Fig. 1C). These conditions were designed to investigate the effect of body size and associated locomotory patterns on leadership. In the Control condition, both fish were allowed to swim in the same tank (Supplemental Information: Video S1). In the Match condition, a small fish swam in a tank with a large replica that replicated the motion of a large fish, which was swimming in the other tank with a small replica that, in turn, replicated the motion of the small fish (Supplemental Information: Video S2). The Match condition aimed at testing whether transferring information from a live individual to robotic replica would preserve the interaction between the live fish observed in the Control condition (a large and a small fish of the same sex swimming together). Should the replicas be appraised as conspecifics, the Match condition would result in a remote interaction between fish in different tanks analogous to the interaction between fish in the same tank of the Control condition. In the Mismatch condition, a small fish swam in a tank with a small replica that replicated the motion of a large fish, which was swimming in the other tank with a large replica that, in turn, replicated the motion of the small fish (Supplemental Information: Video S3). The Mismatch condition was intended to decouple body size from locomotory patterns, thereby introducing a manipulation in the remote interaction between fish.

At the beginning of each experiment, each of the fish in the pair was placed in a separate opaque cylinder in the experimental tanks and allowed to habituate to the water temperature for ten minutes. At the end of the habituation phase, the cylinders were lifted, and the manipulators and cameras were turned on. After ten minutes of experiment, the fish were placed back into their housing tanks in pairs.

Data analysis

Data were analyzed using the statistical toolbox in Matlab R2019b. First, the trajectories obtained from live tracking at a sampling rate of 20 frames per second were smoothed using a Gaussian weighted moving average over a 0.5 s window. To quantify the ability of the replicas to mirror the motion of the fish, we introduced a similarity index as a normalized two-dimensional cross-correlation of the trajectories of the replica and the corresponding live fish in the opposite tank (Fig. 2A). First, we divided the data into time-intervals of 20-s with a total of n=400 data points. Then, cross-correlation between the position of the replica and the fish over the k-th time-interval (each of 20-s) at a time-lag of τ time-steps (each of 0.05 s in duration) was computed as (Buck, 2002)

$$C(\tau,k)=\sum_{t=1}^{n-\tau} R(t+\tau+(k-1)n)\cdot F(t+(k-1)n)$$

,

where a dot is used to indicate inner product between the two-dimensional position of the replica and the fish, R and F. The computed cross-correlation value was normalized by the autocorrelation of each process through

$$\hat{C}(\tau,k) = \frac{C(\tau,k)}{\sqrt{\sum_{t=1}^{n}\|R(t+(k-1)n)\|^2}\sqrt{\sum_{t=1}^{n}\|F(t+(k-1)n)\|^2}}$$

where we have used $\|\cdot\|$ to identify the Euclidean norm of a vector. The normalized cross-correlation ranges between 0 and 1, with 1 corresponding to the replica exactly mirroring the two-dimensional position of the fish at a time-lag τ for the entire duration of the k-th time-interval.

By maximizing the normalized cross-correlation as a function of the time-lag, we gathered information regarding the physical delay in transferring information between the tanks. At the same time, the corresponding value of the maximum normalized cross-correlation is indicative of the accuracy of the robotic platform in replicating the motion of a live animal. Hence, we defined a similarity index as the maximum value of the normalized cross-correlation and we retained the time-lag at which such a maximum was attained for all the considered time-intervals. We deemed successful the trials in which the average similarity index was more than 0.95 and the average time-lag at which the cross-correlation was maximized was less than 0.2 s, for both the instances of behavioral teleporting that were conducted in parallel in the two tanks. Each experimental trial consisted of thirty time-intervals of 20 s. Based on the similarity index results, we identified two trials, one for Match and one for Mismatch conditions, where the replicas failed to replicate (similarity index below 95% within a time-lag of up to 0.2 s) fish motion for more than 70% of the time-intervals. Excluding these two trials, the robotic replicas were successful in replicating fish motion in 85% of the experimental time (Fig. 2B).

Building on our previous work (Porfiri, 2018), we used information-theoretic methods to determine influence between small and large zebrafish. Transfer entropy measures the reduction of the uncertainty in the prediction of the future of a dynamical system from its past, given additional information regarding the past of another dynamical system. A non-zero transfer entropy value points at a possible causal influence of a dynamical system on another in a Wiener-Granger sense (Bossomaier et al., 2016).

To score transfer entropy between the fish, we employed a symbolic approach (Porfiri, 2018), where the time-series of the fish were mapped to discrete symbols. First, we down-sampled the time-series of speed and turn rate of each fish at 10 Hz, to reduce the effect of noise, following standard practice in transfer entropy analysis (Porfiri, 2018) (Supplemental Information: Text S4 and Fig. S6). Speed was measured as the magnitude of the first-order numerical derivative of the position obtained from tracking, and turn rate was calculated with a finite difference approximation of the curvature of the trajectory. Then, we jointly symbolized the time-series based on ordinal patterns, which would encapsulate a simplified ethogram of each animal. For each time-series, we used binary symbols, associated with increasing and decreasing trends of consecutive values (Fig. 4A,B). Then, we merged the two time-series of binary symbols into one time-series of four possible symbols, each representing a combination of increasing/decreasing trend in speed and turn rate (Fig. 4C).

We computed the transfer entropy from small to large fish at a given delay of τ time-steps (each of 0.1 s in duration) as follows:

$$\mathrm{TE}_{\mathrm{Small}\to\mathrm{Large}} = \sum_{L(t+1),L(t),S(t-\tau)} \Pr(L(t+1),L(t),S(t-\tau))\log_2 \frac{\Pr(L(t+1)|L(t),S(t-\tau))}{\Pr(L(t+1)|L(t))}$$

where L and S are the symbolized time-series for large and small fish, “Pr” is the probability mass function computed with a plug-in estimation, over the 20-s time-intervals where the similarity index was above 0.95 for a time-lag less than 0.2 s, and τ is the delay. No time-delay was used for the Control condition as the two fish interacted in real time, and a time-delay of 0.2 s ($\tau = 2$) was used for the Match and Mismatch conditions, to account for the physical delays in replicating the motion of a fish on the replica in the opposite tank (Supplemental Information: Text S4 and Fig. S6). Transfer entropy from large to small fish was computed by simply exchanging L and S the equation above.

We scored the significance of the information transfer using a permutation test (Nichols and Holmes, 2002). More specifically, we computed transfer entropy values between all possible shuffling of trials (*N)* of a condition. From this $N \times N$ matrix, *N* transfer entropy values were randomly selected, and their average values were recorded. This process was repeated for a total of 20,000 times to create a surrogate distribution, which was used to test the null hypothesis. To infer an influence through transfer entropy, we statistically tested true transfer entropy values against the surrogate distribution with a permutation test at 5% significance level.

Cross-correlation analysis was used as an additional measure to further investigate leadership within the pair, thereby isolating whether a fish would tend to initiate maneuvers that will be followed by the other (Krause et al., 2000). First, the speed and turn rate of live fish were down-sampled to 10 Hz, similar to the transfer entropy analysis. Then, we calculated the maximum cross-correlation and corresponding time-lag for both speed and turn rates of two live fish over the 20-s time-intervals with similarity scores above 0.95 for a time-lag less than 0.2 s, independently. We adapted the formula for the similarity index, by simply using one-dimensional time-series (speed or turn rate) of the two fish in place of the two-dimensional position data of the fish and replica. In this vein, a large value of the maximum normalized cross-correlation of any of these two metrics would identify coordination between the fish and the sign of the corresponding time-lag would reveal which fish acts as a leader. Similar to the transfer entropy analysis, the time-series for Match and Mismatch conditions were offset of 0.2 s to account for the platform delay. For example, a time-lag of 0.1 s from small to large fish in Match condition would mean that the small fish led the large fish by 0.3 s. We created a surrogate dataset for each of the metric (maximum value of normalized cross-correlation and corresponding time-lag for both speed and turn rate) by using the same approach as described for transfer entropy, and we analogously assessed statistical significance using a permutation test at 5% significance level.

Ethics statement

All animal procedures were approved by the University Animal Welfare Committee of New York University under protocol number 13-1424.

Supplemental references

Blackman, S.S. (1986). Multi-target tracking with radar applications (Dedham, MA: Artech House, Inc.).

Bonnet, F., Kato, Y., Halloy, J., and Mondada, F. (2016). Infiltrating the zebrafish swarm: design, implementation and experimental tests of a miniature robotic fish lure for fish–robot interaction studies. Artificial Life and Robotics *21*, 239-246.

Bossomaier, T., Barnett, L., Harré, M., and Lizier, J.T. (2016). An introduction to transfer entropy. Cham: Springer International Publishing, 65-95.

Buck, J.R. (2002). Computer explorations in signals and systems using MATLAB, 2nd edn (Upper Saddle River, N.J. :: Prentice Hall).

Butail, S., Mwaffo, V., and Porfiri, M. (2016). Model-free information-theoretic approach to infer leadership in pairs of zebrafish. Physical Review E *93*, 1-12.

Collignon, B., Séguret, A., Chemtob, Y., Cazenille, L., and Halloy, J. (2019). Collective departures and leadership in zebrafish. PLoS ONE *14*, 1-16.

De Lellis, P., Cadolini, E., Croce, A., Yang, Y., di Bernardo, M., and Porfiri, M. (2020). Model-based feedback control of live zebrafish behavior via interaction with a robotic replica. IEEE Transactions on Robotics *36*, 28-41.

Jeon, S.K. (2011). An open source, embedded, high performance g-code-parser and CNC milling controller written in optimized C that will run on a straight Arduino (GitHub).

Kim, C., Ruberto, T., Phamduy, P., and Porfiri, M. (2018). Closed-loop control of zebrafish behaviour in three dimensions using a robotic stimulus. Scientific Reports *8*, 657-657.

Krause, J., Hoare, D., Krause, S., Hemelrijk, C., and Rubenstein, D. (2000). Leadership in fish shoals. Fish and Fisheries *1*, 82-89.

Landgraf, T., Bierbach, D., Nguyen, H., Muggelberg, N., Romanczuk, P., and Krause, J. (2016). RoboFish: Increased acceptance of interactive robotic fish with realistic eyes and natural motion patterns by live Trinidadian guppies. Bioinspiration and Biomimetics *11*.

Miller, N., and Gerlai, R. (2012). From schooling to shoaling: patterns of collective motion in zebrafish (*Danio rerio*). PLoS ONE *7*, e48865.

Munkres, J. (1957). Algorithms for the assignment and transportation problems. Journal of the Society for Industrial and Applied Mathematics *5*, 32-38.

Nichols, T.E., and Holmes, A.P. (2002). Nonparametric permutation tests for functional neuroimaging: A primer with examples. Human Brain Mapping *15*, 1-25.

Oliveira, J., Silveira, M., Chacon, D., and Luchiari, A. (2015). The zebrafish world of colors and shapes: preference and discrimination. Zebrafish *12*, 166-173.

Porfiri, M. (2018). Inferring causal relationships in zebrafish-robot interactions through transfer entropy: a small lure to catch a big fish. Animal Behavior and Cognition *5*, 341-367.

Romano, D., Benelli, G., Hwang, J.-S., and Stefanini, C. (2019). Fighting fish love robots: mate discrimination in males of a highly territorial fish by using female-mimicking robotic cues. Hydrobiologia *833*, 185-196.

Romano, D., Elayan, H., Benelli, G., and Stefanini, C. (2020). Together We Stand–Analyzing Schooling Behavior in Naive Newborn Guppies through Biorobotic Predators. Journal of Bionic Engineering *17*, 174-184.

Strandburg-Peshkin, A., Twomey, C.R., Bode, N.W., Kao, A.B., Katz, Y., Ioannou, C.C., Rosenthal, S.B., Torney, C.J., Wu, H.S., Levin, S.A., *et al.* (2013). Visual sensory networks and effective information transfer in animal groups. Current Biology *23*, R709-R711.

Tunstrøm, K., Katz, Y., Ioannou, C.C., Huepe, C., Lutz, M.J., and Couzin, I.D. (2013). Collective states, multistability and transitional behavior in schooling fish. PLoS Computational Biology *9*, e1002915.

Weber, I., Florin, E., Von Papen, M., and Timmermann, L. (2017). The influence of filtering and downsampling on the estimation of transfer entropy. PLoS ONE *12*, e0188210.

Wibral, M., Pampu, N., Priesemann, V., Siebenhühner, F., Seiwert, H., Lindner, M., Lizier, J.T., and Vicente, R. (2013). Measuring information-transfer delays. PLoS ONE *8*, e55809.
